# Total hip replacement infected with *Mycobacterium tuberculosis *complicated by Addison disease and psoas muscle abscess: a case report

**DOI:** 10.1186/1752-1947-6-3

**Published:** 2012-01-10

**Authors:** Pasquale De Nardo, Angela Corpolongo, Aristide Conte, Elisa Gentilotti, Pasquale Narciso

**Affiliations:** 1 Clinical Department, National Institute for Infectious Diseases 'L. Spallanzani', via Portuense 292, 00149 Rome, Italy

## Abstract

**Introduction:**

Prosthetic joint infection due to *Mycobacterium tuberculosis *is occasionally encountered in clinical practice. To the best of our knowledge, this is the first report of a prosthetic joint infection due to *Mycobacterium tuberculosis *complicated by psoas abscesses and secondary Addison disease.

**Case presentation:**

A 67-year-old immunocompetent Caucasian woman underwent total left hip arthroplasty because of osteoarthritis. After 18 months, she underwent arthroplasty revision for a possible prosthetic infection. Periprosthetic tissue specimens for bacteria were negative, and empirical antibiotic therapy was unsuccessful. She was then admitted to our department because of complications arising 22 months after arthroplasty. A physical examination revealed a sinus tract overlying her left hip and skin and mucosal pigmentation. Her levels of C-reactive protein, basal cortisol, adrenocorticotropic hormone, and sodium were out of normal range. Results of the tuberculin skin test and QuantiFERON-TB Gold test were positive. Computed tomography revealed a periprosthetic abscess and the inclusion of the left psoas muscle. Results of microbiological tests were negative, but polymerase chain reaction of a specimen taken from the hip fistula was positive for *Mycobacterium tuberculosis*. Our patient's condition was diagnosed as prosthetic joint infection and muscle psoas abscess due to *Mycobacterium tuberculosis *and secondary Addison disease. She underwent standard treatment with rifampicin, ethambutol, isoniazid, and pyrazinamide associated with hydrocortisone and fludrocortisone. At 15 months from the beginning of therapy, she was in good clinical condition and free of symptoms.

**Conclusions:**

Prosthetic joint infection with *Mycobacterium tuberculosis *is uncommon. A differential diagnosis of tuberculosis should be considered when dealing with prosthetic joint infection, especially when repeated smears and histology examination from infected joints are negative. Clinical outcomes of prosthetic joint infection by *Mycobacterium tuberculosis *are unpredictable, especially given the limited literature in this field and the uncertainty of whether medical treatment alone can eradicate the infection without prosthesis removal. Furthermore, this case report raises interesting issues such as the necessity of a follow-up evaluation after treatment based on clinical conditions, the utility of a more standardized length of treatment for periprosthetic tuberculous infection, and the importance of a high diffusion capacity of anti-mycobacterial agents in order to eradicate the infection.

## Introduction

The total number of individuals living with primary total hip replacement has increased in recent years [1]. The majority of prosthetic joints result in successful outcomes. Complications due to prosthetic joint infections (PJIs) are seen in 0.3% to 1.7% of hip arthroplasties [2-4]. The most common agents of PJI are staphylococci, which are responsible for more than half of the cases. Occasionally, infections due to rare organisms such as *Mycobacterium *species may occur [5].

Thomas Addison first described the clinical and pathological features of adrenal failure more than 150 years ago. At that time, the major cause of adrenal failure was bilateral adrenal destruction due to *Mycobacterium *(MTB) infection. After the development of effective anti-tuberculosis chemotherapy, the incidence of Addison disease due to tuberculosis decreased [6]. We report a case of PJI due to MTB complicated by psoas abscesses and secondary Addison disease.

## Case presentation

A 67-year-old Caucasian woman was seen at our clinic for possible prosthetic infection following a total left hip replacement. Her medical history was negative until three years before, when she began to complain of left hip pain during walking, movement limitation, and pain at rest. After one year of unsuccessful prior rehabilitative and analgesic treatment, she underwent total left hip replacement. The results of a pre-operative chest X-ray and blood tests were normal; however, erythrocyte sedimentation rate (ESR) was elevated (94 mm/hour). The post-operative period was uneventful, and the patient was discharged five days after surgery. She remained in good clinical condition until about 16 months after surgery, when she began to feel pain and movement limitation at the prosthetic site. Two months later, a hip computed tomography (CT) scan showed a suspect iliopsoas abscess, which was confirmed by peripheral blood mononuclear cells marked with ^99 m^Tc. Her clinical conditions worsened because of increasing local pain and an irregular mild fever. She was admitted to an orthopedic unit for needle aspiration of the abscess (102 × 53 mm) under ultrasound guidance. Culture of the needle aspiration and blood cultures for aerobic and anaerobic bacteria were performed, and empiric antibiotic therapy was started with intravenous linezolid (600 mg twice a day). Her fever increased, and all cultures were negative. After one week of unsuccessful medical treatment, the surgeon decided to perform surgery to inspect and clean the infected site.

A pseudocyst containing dense material was found just near the prosthesis. A complete aspiration with culture of dense and cloudy liquid, including biopsies of the wall of the cyst and the surrounding tissue, was performed. Intravenous antibiotic therapy with 400 mg of teicoplanin, 160 mg of gentamicin, and 800 mg of ciprofloxacin was started. After four days, the fever disappeared and the patient was discharged without antibiotic therapy. Repeat cultures were negative. A histopathological examination disclosed a fibrous tissue with acute and chronic inflammation, partly with an aspecific granulomatous aspect.

Twenty days later, the patient complained of mild fever and a painful, fluctuant nodule located at the superior internal surface of the left thigh along the surgical scar. The nodule was then surgically drained. A large quantity of cloudy liquid was collected from the site. Cultures were again negative. At the site of the cut, there appeared to be a deep fistula of about 5 cm in diameter with discrete, greenish, and odorless secretion. There was no evidence of inflammation to the surrounding tissues. The patient was then admitted to our unit. Her vital signs were normal. During a physical examination, she showed bad general health conditions and evident brownish coloration of the skin to the extremities (hands, feet, palmar, and plantar plicae), nipples, linea alba, and oral mucosa. The cutaneous fistula appeared unchanged. Laboratory studies showed the following: red blood cell count of 4.53 × 109/L, hemoglobin of 12.9 g/dL, platelet count of 336 × 109/L, white blood cell count of 8.2 × 109/L (60.9% neutrophilis and 24.2% lymphocytes), ESR of 89 mm/hour, and C-reactive protein (CRP) of 75 mg/L (normal value is less than 0.5 mg/L). The result of a tuberculin skin test (5 IU purified protein derivative) was positive, QuantiFERON-TB Gold was 3.08 IU/mL (cutoff value is 0.35 IU/mL), serum cortisol at 8 a.m. was 9.5 μg/dL (normal value is 4.3 to 22.4), adrenocorticotropic hormone (ACTH) was 2300 pg/mL (normal value is 10 to 60), and anti-cortex antibodies were negative. The second controls of serum cortisol and ACTH were 5.5 μg/dL and 1490 pg/mL, respectively. Major causes of adrenal insufficiency are autoimmune disorders and MTB infection. Anamnesis, CT scan of adrenal glands, and the absence of anti-cortex antibodies suggested exclusion diagnosis of Addison disease secondary to MTB infection. The result of an experimental whole blood test based on interferon-gamma (IFNγ) response to RD1-selected peptides, which tends to be related to active tuberculosis, was also positive [7]. The active IFNγ response was 2.6 IU/mL (cutoff is 0.7 IU/mL). The remaining biochemical parameters were normal. The amount of secretion was discrete; thus, fistula microbiological cultures could not be done. A single specimen was collected to perform Gram and Ziehl-Neelsen staining. Only polymerase chain reaction (PCR) for MTB complex was positive.

**Figure 1:**
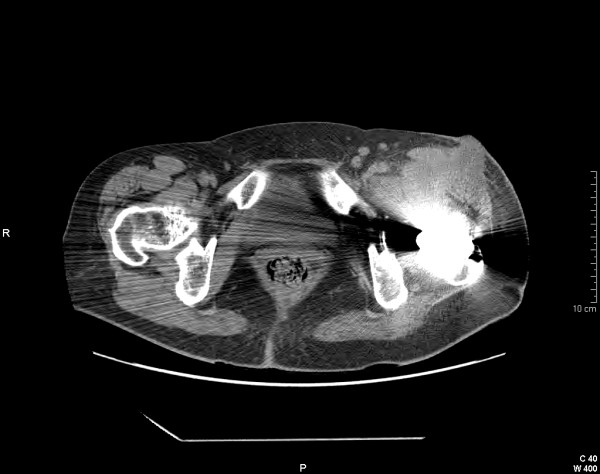
**A computed tomography scan image of hip arthroplasty**. Evidence of a periprosthetic abscess with diffusion to the left psoas muscle and cutaneous fistula is shown.

Hip, abdominal, and chest CT scans revealed a periprosthetic abscess with diffusion to the left psoas muscle (Figure 1), a slight bilateral adrenal enlargement with calcifications, and a mild fibrosis of both upper lobes of the lungs. Our patient's condition was diagnosed on the basis of the laboratory and clinical findings as tuberculosis and secondary Addison disease. So that Pott disease could be excluded, our patient underwent a backbone X-ray, which was negative. She was put on treatment with anti-tuberculosis agents: rifampicin (RFP), ethambutol, isoniazid (INH), pyrazinamide, and pyridoxine without removal of the prosthesis. Hydrocortisone and fludrocortisone were prescribed for Addison disease. After three months, the anti-tuberculosis treatment was continued with RFP and INH only. The fistula continued to slowly discharge discrete amounts of secretion and appeared to be completely healed after six months. Our patient was totally asymptomatic and without pain upon hip movements after 15 months. The iliopsoas abscess was no longer evident on a CT scan performed about one year later.

## Discussion

Infections may occur after hip arthroplasties in 0.3% to 1.7% of cases. The most common agents are Gram-positive cocci (in approximately 65% of cases) (*Staphylococcus aureus*, Coagulase-negative *Staphylococcus *species, and *Enterococcus *species), aerobic Gram-negative bacilli (approximately 6%), anaerobes (4%, especially in shoulder arthroplasties), polymicrobial (20%), and fungi (1%). Cultures are negative in 7% of cases [8]. Infections can be classified according to when the clinical symptoms appear after surgery: early (less than three months), delayed (three to 24 months), or late (more than 24 months after the operation) [9].

In our case study, all typical microbiological tests, including those specimens obtained during surgery, were always negative. This is not commonly seen in patients with chronic prosthetic infections, especially in the presence of cutaneous fistulae, in which cultures tend to be positive, sometimes showing mixed or unusual agents. These factors render diagnosis and treatment difficult.

In spite of the current advances in diagnostic technology, a physical examination of the patient remains of utmost importance in determining the correct clinical diagnosis. The presence of skin and mucosal pigmentation linked to negative microbiological cultures was a key finding in our patient in the differential diagnosis of MTB infection with secondary adrenal failure.

The medical history of our patient revealed interesting findings. She reported periods of dry cough, mild fever at night, and weight loss at the age of six. Symptoms spontaneously regressed after about two months. Owing to socioeconomic reasons, our patient did not undergo clinical examinations. The hospital hip replacement medical chart did not show any strange or abnormal clinical findings; however, our patient recalled that the surgeon mentioned that, during surgery, there were difficulties due to the presence of a hard intra-articular white tissue. Unfortunately, histological studies were not performed at that time. The clinical and CT scan findings of our patient, in the presence of Addison disease, gave rise to the differential diagnosis of a chronic, symptomless, tuberculosis infection. At the time of surgery, our patient could have already had MTB coxitis. Furthermore, a granulomatous aspect in tissue samples from periprosthetic tissue in the absence of a detectable foreign body reaction is a strong indication to evaluate for tuberculosis infection. The gold standard in the diagnosis of tuberculosis remains a positive MTB cultural test.

**Figure 2:**
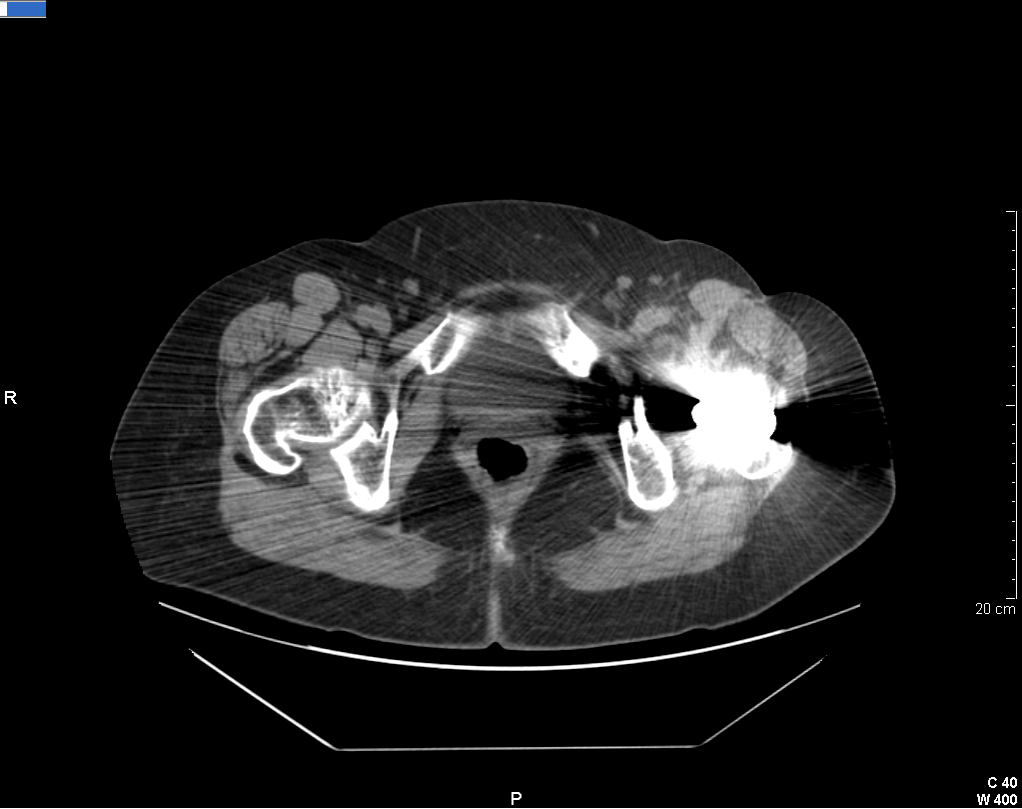
**An image of a computed tomography scan performed 12 months after treatment**. Regression of the iliopsoas abscess is shown. The cutaneous fistula is completely healed.

Current technology provides newer and faster testing to confirm the clinical suspicion of MTB infection. PCR of biological specimens is one of the most useful, rapid, and specific tests currently available. PCR can be performed even in formaline-embedded tissue samples and has good sensitivity [10]. In addition, owing to issues in sensitivity and specificity, swabs from sinus tracts are not recommended. The current scientific literature in this field is geared toward new laboratory methods to detect active infections; the test based on IFNγ response to RD1-selected peptides is just one example. The global clinical health conditions of our patient improved over time. She put on some weight, her skin was slowly discoloring back to normal, she was walking correctly without pain, and the cutaneous fistula completely healed. The CT scan confirmed the regression of the iliopsoas abscess about 12 months after treatment (Figure 2). Our patient is currently continuing the oral regime of RFP and INH to complete at least 18 months of treatment. According to a widely accepted algorithm published by Zimmerli and colleagues [11], healing of the infection can be assumed only if the patient has no signs of recurrence for at least two years after interruption of anti-tuberculosis treatment. Furthermore, because the soft tissues were heavily damaged and because MTB can be considered difficult-to-treat bacteria, no retention of implant was suggested. Hence, management of this PJI should have included a two-stage exchange with an interval of six to eight weeks between removal of the first prosthesis and placement of the second, without the use of a spacer [11].

## Conclusions

This case study raises the following important issues:

• It is well known that bacteria, in addition to MTB, are capable of forming and surviving within the biofilm found on the surface of prosthetic materials and thus are protected from immunological and antibiotic response. It was not clear in our patient whether her hip prosthesis had been colonized by MTB protected within the biofilm. PJI should still be suspected, even when routine cultures from infected joints are negative.

• Follow-up evaluations after treatment should be based on clinical conditions, ESR, CRP, and radionuclide study.

• Recurrent infection poses difficulties in surgical removal of the implant. The distal part of the prosthesis tends to be stabilized on the femur diaphysis, rendering surgical removal very dangerous and traumatic.

• There are several open questions in medical and surgical treatment of the total hip replacement infected with MTB (in regard to choice of medical therapy, duration, complications, and so on). The length of treatment for periprosthetic tuberculosis infection is debatable and not standardized. The medical drug treatment in our patient included four drugs for two months, followed by 16 months of therapy with only two drugs, which provided good results. The anti-mycobacterial agents used have a high diffusion capacity, which probably allowed eradication of the infection.

A more standardized assessment of diagnosis methods and medical and surgical options for the treatment of a total hip replacement infected with MTB appears to be desirable in order to help clinicians improve the quality of care.

## Consent

Written informed consent was obtained from the patient for publication of this case report and any accompanying images. A copy of the written consent is available for review by the Editor-in-Chief of this journal.

## Abbreviations

ACTH: adrenocorticotropic hormone; CRP: C-reactive protein; CT: computed tomography; ESR: erythrocyte sedimentation rate; IFNγ: interferon-gamma; MBT: *Mycobacterium tuberculosis*; PCR: polymerase chain reaction; PJI: prosthetic joint infection.

## Competing interests

The authors declare that they have no competing interests.

## Authors' contributions

PDN, ACor, ACon, and PN (infectious disease specialists) were the reference physicians during the in-hospital management of this case. All authors contributed to the drafting of the article and read and approved the final manuscript.
